# Beyond the Mean: Quantile Regression to Explore the Association of Air Pollution with Gene-Specific Methylation in the Normative Aging Study

**DOI:** 10.1289/ehp.1307824

**Published:** 2015-03-13

**Authors:** Marie-Abele C. Bind, Brent A. Coull, Annette Peters, Andrea A. Baccarelli, Letizia Tarantini, Laura Cantone, Pantel S. Vokonas, Petros Koutrakis, Joel D. Schwartz

**Affiliations:** 1 Department of Environmental Health, and; 2 Department of Biostatistics, Harvard T.H. Chan School of Public Health, Boston, Massachusetts, USA; 3 Institute of Epidemiology, Helmhotz Zentrum München–German Research Center for Environmental Health, Neuherberg, Germany; 4 Department of Clinical Sciences and Community Health, Center of Molecular and Genetic Epidemiology, Università degli Studi di Milano, Milan, Italy; 5 Fondazione Cà Granda, IRCCS (Istituto Di Ricovero e Cura a Carattere Scientifico) Ospedale Maggiore Policlinico, Milan, Italy; 6 VA Normative Aging Study, VA Boston Healthcare System, Boston, Massachusetts, USA; 7 Department of Medicine, Boston University School of Medicine, Boston, Massachusetts, USA

## Abstract

**Background:**

Air pollution has been related to mean changes in outcomes, including DNA methylation. However, mean regression analyses may not capture associations that occur primarily in the tails of the outcome distribution.

**Objectives:**

In this study, we examined whether the association between particulate air pollution and DNA methylation differs across quantiles of the methylation distribution. We focused on methylation of candidate genes related to coagulation and inflammation: coagulation factor III (*F3*), intercellular adhesion molecule 1 (*ICAM-1*), interferon gamma (*IFN-***γ**), interleukin-6 (*IL-6*), and toll-like receptor 2 (*TRL-2*).

**Methods:**

We measured gene-specific blood DNA methylation repeatedly in 777 elderly men participating in the Normative Aging Study (1999–2010). We fit quantile regressions for longitudinal data to investigate whether the associations of particle number, PM_2.5_ (diameter ≤ 2.5 μm)black carbon, and PM_2.5_ mass concentrations (4-week moving average) with DNA methylation [expressed as the percentage of methylated cytosines over the sum of methylated and unmethylated cytosines at position 5 (%5mC)] varied across deciles of the methylation distribution. We reported the quantile regression coefficients that corresponded to absolute differences in DNA methylation (expressed in %5mC) associated with an interquartile range increase in air pollution concentration.

**Results:**

Interquartile range increases in particle number, PM_2.5_ black carbon, and PM_2.5_ mass concentrations were associated with significantly lower methylation in the lower tails of the *IFN-***γ** and *ICAM-1* methylation distributions. For instance, a 3.4-μg/m^3^ increase in PM_2.5_ mass concentration was associated with a 0.18%5mC (95% CI: –0.30, –0.06) decrease on the 20th percentile of *ICAM-1* methylation, but was not significantly related to the 80th percentile (estimate: 0.07%5mC, 95% CI: –0.09, 0.24).

**Conclusions:**

In our study population of older men, air pollution exposures were associated with a left shift in the lower tails of the *IFN-***γ** and *ICAM-1* methylation distributions.

**Citation:**

Bind MA, Coull BA, Peters A, Baccarelli AA, Tarantini L, Cantone L, Vokonas PS, Koutrakis P, Schwartz JD. 2015. Beyond the mean: quantile regression to explore the association of air pollution with gene-specific methylation in the Normative Aging Study. Environ Health Perspect 123:759–765; http://dx.doi.org/10.1289/ehp.1307824

## Introduction

Air pollution affects people every day and especially the elderly, who are a growing population stratum in the United States. Mechanisms by which air pollution causes cardiovascular mortality and morbidity are not fully elucidated (Brook et al. 2010). Recent research has pointed to epigenetics as a potential mechanism for the adverse effects of air pollution (Breton et al. 2012; Jardim 2011; Madrigano et al. 2012a). Epigenetics refers to chromosome changes that do not modify the genetic code, but influence its expression. The most frequently examined epigenetic mechanism is called DNA methylation because it involves methylation of cytosine in CpG (cytosine–phosphate–guanine) pairs.

Several studies have related air pollution exposure to changes in epigenetic outcomes, including DNA methylation, but they have used standard regression methods that report the change in the expected value of an outcome for a given change in exposure (Baccarelli et al. 2009; Bellavia et al. 2013; Soberanes et al. 2012; Tarantini et al. 2009). However, focusing on the mean response may not well describe effects that shift the overall shape, versus the location, of the outcome distribution. Because DNA methylation is a biological mechanism whereby cells control gene expression in a complex manner (stochastic dynamics, phase variation, and bistability) (Riggs and Xiong 2004), we hypothesized that mean regression analyses may not capture associations that occur primarily in the tails of the outcome distribution.

In this study, we examined whether air pollution affects DNA methylation across nine quantiles of the methylation distribution. We focused on methylation of candidate genes related to coagulation and inflammation: coagulation factor III (*F3*), intercellular adhesion molecule 1 (*ICAM-1*), toll-like receptor 2 (*TRL-2*), interferon gamma (*IFN-*γ), and interleukin 6 (*IL-6*). Previous research has shown that high levels of similar markers of coagulation and inflammation increase the risk of cardiovascular-related outcomes (Danesh et al. 1998; Hwang et al. 1997; Mendall et al. 1996). We studied a cohort of elderly men who may have greater susceptibility to air pollution exposure because of their age (Shumake et al. 2013).

## Materials and Methods

*Study population*. This prospective cohort study included male participants from the Normative Aging Study, an investigation established in Boston, Massachusetts, in 1963 by the U.S. Veterans Administration (Bell et al. 1966). We measured DNA methylation on blood samples collected after an overnight fast and smoking abstinence during the period 1999–2009. Methylation was assessed using blood samples collected at one to five visits completed at 3- to 5-year intervals. About 70% of the participants had more than one medical visit. We excluded individual observations if C-reactive protein levels were > 10 mg/L (74 observations in 71 participants) to reduce the potential influence of current infections (Simon et al. 2004), leaving a total of 1,798 observations in 777 participants. This study was approved by the Harvard T.H. Chan School of Public Health and the Veterans Administration Institutional Review Boards (IRB). Participants provided written informed consent to participate in this study, which was approved by the Veterans Administration IRB.

*Air pollution*. The relevant exposure window for the association of air pollution with DNA methylation is unknown. Previous studies suggested an association spread over several weeks (Baccarelli et al. 2009; Madrigano et al. 2011; Salam et al. 2012). In the same cohort, we observed some associations between air pollution exposure averaged up to 1 month preceding the medical visit and the mean of the gene-specific methylation distribution (Bind et al. 2014). Therefore, we chose *a priori* to explore a similar intermediate-term exposure window and focused on air pollution concentrations averaged over the monthly period preceding each participant’s methylation assessment. We examined only one exposure window to limit the number of tests. The intermediate time window could be a proxy for short- and long-term exposures.

The exposure variables we considered are 4-week moving averages of particle number concentration (including fine and ultrafine particles 0.007–3 μm in diameter; number/cubic centimeters), PM_2.5_ mass concentration (particles ≤ 2.5 μm in diameter; micrograms per cubic meter), and PM_2.5_ black carbon (black carbon particles ≤ 2.5 μm in diameter; micrograms per cubic meter). Particulate concentrations were measured hourly at the Harvard supersite located near downtown Boston and approximately 1 km from the examination center. Because the study participants lived in the Greater Boston area with a median distance of 20 km from the Harvard supersite, we assumed that the ambient air pollutant concentrations could serve as surrogates of their exposures. We measured hourly particle number in the 0.007- to 3-μm size range with a condensation particle counter (model 3022A; TSI Inc., Shoreview, MN), hourly PM_2.5_ elements with a tapered element oscillation microbalance (model 1400A; Rupprecht and Pastashnick, East Greenbush, NY), and hourly PM_2.5_ black carbon concentrations using an aethalometer (model AE-16; Magee Scientific Co., Berkeley, CA). From the hourly measurements, we calculated 24-hr mean concentrations and then monthly moving averages using the corresponding 4-week lags.

Whereas particle number is a marker for fresh local traffic emissions, PM_2.5_ black carbon originates from both local and transported traffic emissions. In Boston, transported sulfate particles and secondary organic aerosols constitute a large fraction of PM_2.5_ mass (Kang et al. 2010).

*DNA methylation*. We collected each participant’s blood at every visit and isolated DNA to assess gene-specific DNA methylation using quantitative methods based on bisulfite polymerase chain reaction pyrosequencing (Yang et al. 2004). The degree of methylation was expressed as the percentage of methylated cytosines over the sum of methylated and unmethylated cytosines at position 5 (%5mC).

Nine candidate genes that were expressed in leukocytes and plausibly related to heart or lung disease were chosen *a priori* for high precision pyrosequencing analysis as part of a previous study. From those nine candidate genes, we focused on five (*F3*, *ICAM-1*, *IFN-*γ, *TRL-2*, and *IL-6*) whose associated proteins are related to coagulation and inflammatory pathways. We previously examined the mean association between air pollution exposure and methylation of the same set of genes (Bind et al. 2014).

We measured *F3*, *ICAM-1*, *IFN-*γ, and *TRL-2*, methylation levels at two to five CpG positions within each gene’s promoter region and calculated the mean values of the position-specific measurements. *IL-6* methylation was quantified outside the gene’s promoter region. Exact positions within promoter regions, as well as primers and conditions for the assays, have been previously described (Bind et al. 2012).

*Weather variables*. Ambient temperatures and relative humidity were measured at the Boston Logan Airport weather station located 8 km from the study center over the 1999–2010 period. Because study participants lived throughout the metropolitan area, we assumed that the monitored temperature and humidity can serve as surrogates of their exposures.

*Statistical methods*. We investigated whether air pollution levels averaged over the 4-week period before the *j*th visit of participant *i* was associated with the *p*th percentile of the DNA methylation distribution ψ_p_(Y_ij_). Because we had repeated methylation measures for 71% of the participants, we fit quantile regressions for longitudinal data and report the associations on the additive scale (Koenker 2004). This approach can be summarized as below:

ψ_p_(Y_ij_ | A_ij_, C_1ij_ = c_1_, C_2ij_ = c_2_, b_i,p_) = (β_0,p_ + b_i,p_) + β_1,p_ A_ij_ + **β**_2,p_^T^ c_1_ + **β**_3,p_^T^ c_2_, [1]

where

A_ij_, Y_ij_, C_1ij_, and C_2ij_ are the air pollution exposure, DNA methylation, set of confounding variables, and the set of risk factors of participant *i* at the *j*th visit, respectively
ψ_p_(Y_ij_) is the *p*th quantile of the Y_ij_ distribution
b_i,p_ is the random intercept for participant *i* included in the regression model for the *p*th quantile of the methylation distribution
β_k,p_ are the coefficients related to the *p*th quantile regression model (k = 0 to 3)
Variables in bold represent vectors.

In our regression models, the dependent variable was gene-specific DNA methylation. We reported the quantile regression coefficients, which correspond to differences in DNA methylation (expressed in %5mC) associated with an interquartile range increase in air pollution concentration. The alpha level for statistical significance was 0.05. We adjusted for potential time-varying confounders (C_1_) such as temperature, relative humidity, sine and cosine terms as a function of day of the season, and batch of methylation measurement. We also controlled for time-varying factors likely to influence methylation (C_2_) but not exposure, such as age, diabetes, body mass index, smoking status (former, current, vs. never smoker), statin use, as well as percentages of neutrophils and lymphocytes in differential blood count. We included C_2_ in the models for efficiency and blocking any potential backdoor path through unmeasured variables that would be a common cause of air pollution and C_2_ (Greenland et al. 1999)_._ We thus assumed no unmeasured confounding between air pollution and methylation, given the random intercept and the C_1_ covariates (see Supplemental Material, Figure S1).

We checked for nonlinear dose–response relationships between the methylation mean and air pollutant concentrations, temperature, and relative humidity using generalized additive models and cubic splines. We found no deviation from linear dose–response relationships with respect to methylation: Using cubic splines, we observed no significant improvement in fit relative to a linear model (data not shown). We conducted some sensitivity analyses restricting the study population to never and former smokers (i.e., using individual observations for men whose smoking status changed over follow-up time). Moreover, we assumed the missing mechanisms of the exposures and outcomes to be at random conditional on the covariates, and the measurement error of air pollution to be primarily Berkson (Zeger et al. 2000).

Quantile regression does not specify any distribution for the residuals, and hence is distribution free. Moreover, if one takes as their regression coefficient estimates those values that minimize the sum of the absolute values of the residuals instead of the sum of squared residuals, the result is an estimate of covariate effects on the median, instead of the mean, of the outcome distribution. Quantile regression generalizes this approach by weighting the positive and negative residuals differently, which forces the regression line to other percentiles of the distribution.

We compared the quantile regression estimates to the ones obtained by a standard mean regression model. Because three methylation distributions (i.e., *F3*, *ICAM-1*, and *TLR-2*) had a point mass at zero and the residuals’ distribution showed important deviation from a Gaussian density, we assumed a Tweedie distribution (with a log-link) for these outcomes and reported associations on the multiplicative scale. For the other two outcome distributions (i.e., *IFN-γ* and *IL-6* methylation), we assumed a Gaussian distribution for the residuals and presented our results on the additive scale. We fit the following linear mixed-effects models:

Mean model for *F3*, *ICAM-1*, and *TLR-2* (multiplicative scale)

log E[Y_ij_] = (γ_0_ + u_i_) + **γ**_1_ A_ij_ + Σ_k_ γ_4k_ C_kij_ with Y_ij_ ~ Tweedie and u_i_ ~ N(0,σ_u_^2^), [2]

Mean models for *IFN-*γ and *IL-6* (additive scale)

Y_ij_ = (γ_0_ + u_i_) + **γ**_1_ A_ij_ + Σ_k_ γ_4k_ C_kij_ + ε_ij_ with ε_ij_ ~ N(0,σ^2^) and u_i_ ~ N(0,σ_u_^2^), [3]

where A_ij_, Y_ij_, and C_kij_ correspond to the air pollution exposure, DNA methylation, and the set of variables for which we adjusted (i.e., confounders and risk factors) for participant *i* at the *j*th visit, respectively.

We constructed an alternative way of presenting the decile-specific results by illustrating the actual distributional change of *IFN-*γ methylation associated with an interquartile range increase in particle number concentration. We estimated a predicted curve using the quantile regression coefficients and assuming a constant trend within decile intervals.

## Results

*Descriptive statistics*. At baseline, the median age of the study population was 72 years. Also, 27% of the participants were obese (defined as body mass index > 30 kg/m^2^), 14% were diabetics, and only 4% were current smokers. Participants’ characteristics varied according to their total number of visits: Individuals with more visits seemed healthier than participants with fewer visits; that is, at baseline, participants with more medical visits over the study period were less likely to be former smokers, statin users, old, obese, or diabetics (Table 1). Boston has a continental climate with direct influences from the ocean. Although it is mostly cold and dry in winter, it is usually warm and humid in summer. Ambient air pollutants levels in Boston are generally below the U.S. Environmental Protection Agency (EPA) standards. Over the 1999–2009 study period, the 24-hr PM_2.5_ mass concentrations exceeded the daily standard of 35 μg/m^3^ for only 13 days: between June and August 2002. Summary statistics of the weather and air pollution as well as Spearman correlations during the study period are presented in Tables 2 and 3, respectively. A substantial number of measurements of particle number concentrations (i.e., 24%) were missing due to a later acquisition of the condensation particle counter or a lack of recording measurements. PM_2.5_ black carbon was positively correlated with PM_2.5_ mass (ρ = 0.68). We found no statistically significant correlation between other pollutants. Temperature was also negatively correlated with particle number (ρ = –0.69), but positively correlated with PM_2.5_ black carbon (ρ = 0.48) and PM_2.5_ mass (ρ = 0.40). The gene-specific methylation distributions varied according to genes (Table 4). For instance, at baseline, we observed wider methylation distributions of *IFN-γ* (5th and 95th percentiles: 75.4, 91.1) and *IL-6* (5th and 95th percentiles: 25.4, 62.1), compared with that of *F3* (5th and 95th percentiles: 1.0, 4.5), *ICAM-1* (5th and 95th percentiles: 2.2, 8.2), and *TLR-2* (5th and 95th percentiles: 1.5, 5.3).

**Table 1:** Demographic characteristics of the Normative Aging Study participants across visits.

Visits	5th, 50th, 95th percentile			Obese (%)^*a*^	Statin user (%)	Diabetic (%)	Smoking status (%) (never, former, current)
	Age (years)	% of neutrophils	% of lymphocytes				
Baseline (*n* = 777)	62, 72, 84	48, 62, 74	15, 26, 38	27	36	14	29, 67, 4
*n*_missing_	0	22	22	0	0	0	0
Among participants having one visit (*n*_1_ = 221)
Visit 1	64, 76, 88	48, 63, 77	13, 25, 37	30	40	18	26, 70, 4
Among participants having two visits (*n*_2_ = 217)
Visit 1	60, 73, 83	47, 62, 74	15, 25, 40	28	35	16	26, 69, 5
Visit 2	66, 77, 86	48, 64, 75	14, 24, 37	27	54	19	26, 70, 4
Among participants having three visits (*n*_3_ = 216)
*Visit 1*	62, 71, 82	47, 62, 72	16, 26, 39	25	36	9	29, 68, 3
*Visit 2*	66, 74, 86	48, 62, 74	15, 26, 38	26	52	13	28, 69, 3
*Visit 3*	69, 78, 89	48, 62, 76	13, 25, 39	25	62	17	27, 71, 2
Among participants having four visits (*n*_4_ = 120)
Visit 1	60, 69, 77	49, 61, 74	15, 26, 36	22	29	10	38, 58, 4
Visit 2	63, 72, 81	46, 62, 78	13, 25, 40	22	42	11	38, 58, 4
Visit 3	66, 75, 84	47, 61, 76	13, 26, 37	18	59	16	38, 59, 3
Visit 4	70, 78, 87	50, 63, 76	12, 25, 37	17	65	18	38, 60, 2
Three individuals had 5 visits, and their characteristics were fairly healthier than those of the other participants. ^***a***^Body mass index > 30 kg/m^2^.							

**Table 2:** Summary statistics for the weather and air pollution variables (4-week moving average).

Variable	*n*_observations_	*n*_missing_	IQR	Percentile		
				5th	50th	95th
Temperature (°C)	1,798	0	13	–1	14	23
Relative humidity (%)	1,798	0	8	58	69	77
Particle number (number per cm^3^)	1,365	433	14,599	9,352	18,426	42,291
PM_2.5_ black carbon (μg/m^3^)	1,798	0	0.26	0.46	0.74	1.04
PM_2.5_ mass (μg/m^3^)	1,798	0	3.4	6.3	9.6	15.1

**Table 3:** Spearman correlations for the weather and air pollution variables (4-week moving average).

Variable	Temperature	Relative humidity	Particle number	PM_2.5_ black carbon	PM_2.5_ mass
Temperature	1	0.41	–0.69	0.48	0.40
Relative humidity		1	–0.05	0.55	0.24
Particle number			1	–0.07	0.07
PM_2.5_ black carbon				1	0.68
PM_2.5_ mass					1

**Table 4:** Gene-specific methylation (%) across visits (5th, 50th, and 95th percentiles).

Visits	*F3* mean	*ICAM-1* mean	*IFN-*γ mean	*TLR-2* mean	*IL-6* mean
*n*_observations_	1,533	1,424	1,736	1,424	1,749
*n*_missing_	265	374	62	374	49
Baseline (*n* = 777)	1.0, 2.0, 4.5	2.2, 4.1, 8.2	75.4, 85.2, 91.1	1.5, 2.8, 5.3	25.4, 43.7, 62.1
Among participants having one visit (*n*_1_ = 221)
Visit 1	1.1, 1.9, 3.5	2.6, 4.3, 7.7	72.4, 85.2, 91.8	1.4, 2.8, 5.0	23.7, 43.8, 61.6
Among participants having two visits (*n*_2_ = 217)
Visit 1	1.0, 2.0, 4.2	2.2, 4.1, 8.4	75.4, 85.5, 90.9	1.5, 2.6, 5.1	23.7, 43.1, 65.3
Visit 2	0.8, 2.3, 4.4	2.2, 3.9, 8.2	75.8, 86.2, 91.4	1.0, 2.6, 5.7	24.7, 42.8, 59.8
Among participants having three visits (*n*_3_ = 216)
Visit 1	1.0, 2.0, 4.5	2.1, 3.8, 7.6	75.8, 84.7, 91.1	1.3, 2.8, 5.2	28.9, 43.7, 59.8
Visit 2	0.9, 2.5, 4.5	2.1, 3.6, 7.8	76.4, 86.8, 90.7	1.5, 2.6, 5.3	28.4, 43.0, 57.5
Visit 3	0.9, 1.8, 4.3	2.9, 4.2, 6.7	76.3, 86.2, 91.1	0.9, 2.1, 4.9	24.9, 42.9, 59.7
Among participants having four visits (*n*_4_ = 120)
Visit 1	0.4, 2.3, 5.2	2.1, 4.0, 9.8	76.9, 84.4, 90.7	1.9, 3.3, 5.9	28.9, 43.8, 61.8
Visit 2	1.0, 2.4, 4.8	2.0, 3.3, 9.9	76.9, 85.6, 91.4	1.7, 3.1, 6.0	25.3, 43.4, 58.4
Visit 3	1.8, 2.9, 4.5	2.5, 4.4, 6.1	75.0, 86.4, 89.3	1.5, 3.0, 6.3	28.7, 44.4, 62.9
Visit 4	0.7, 1.3, 3.1	2.8, 4.0, 8.3	77.5, 86.2, 92.7	0.9, 1.6, 4.0	26.3, 44.9, 60.5
This table does not include three individuals having five visits.					

**Figure 1:**
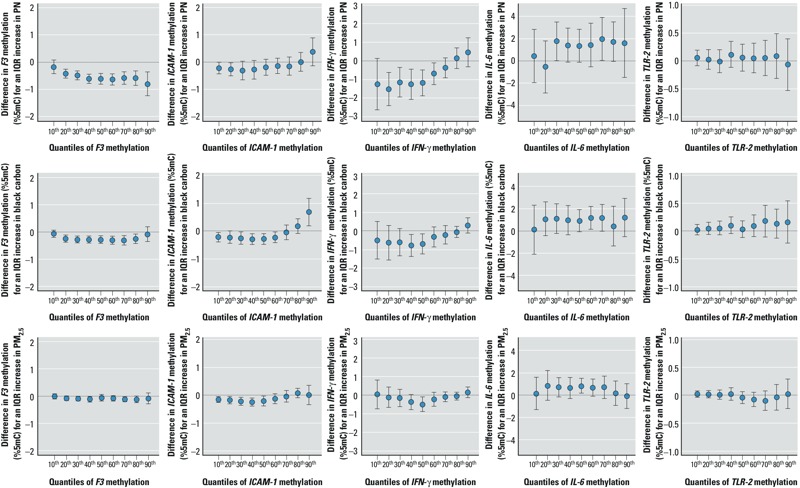
Absolute difference in gene-specific methylation (expressed in %5mC with 95% CI) associated with an IQR increase in exposure [interquartile range (IQR) = 14,599 per cm^3^ for particle number (PN), 0.26 μg/m^3^ for PM_2.5_ black carbon, and 3.4 μg/m^3^ for PM_2.5_ mass], according to the deciles of the methylation distribution.

*Quantile regression results*. Our results showed that the air pollution association with DNA methylation was not generally homogeneous across quantiles (Figure 1). We observed that the negative association between particle number and *F3* methylation was stronger in the upper deciles of the *F3* methylation distribution. Although concentrations of particle number and PM_2.5_ were not associated with *ICAM-1* methylation in the upper deciles of the methylation distribution, they were negatively related to the lowest deciles. For instance, a 3.4-μg/m^3^ increase in PM_2.5_ was associated with a 0.18%5mC [95% confidence interval (CI): –0.30, –0.06] decrease on the 20th quantile of *ICAM-1* methylation, and was not significantly related to the 80th quantile (estimate: 0.07%5mC; 95% CI: –0.09, 0.24). PM_2.5_ black carbon concentrations were negatively associated with the 10th to 60th percentiles of the *ICAM-1* methylation distribution and positively related to the 90th percentile. Moreover, we observed that the negative association between particle number and *IFN-*γ methylation was strongest in the lower deciles of the *IFN-*γ methylation distribution. Particle number concentrations were not related to the 10th and 20th percentiles but were associated with the higher deciles of the *IL-6* methylation distribution. We did not find any associations between air pollution and any of the deciles of the *TLR-2* methylation distribution. In the analysis restricted to never and former smokers (consisting of 755 participants and 1,737 individual observations), we found fairly similar results (see Supplemental Material, Figure S2).

The results obtained from mean regression analyses are presented in Table 5. An interquartile range increase in particle number concentration was negatively associated with the means of the *F3* and *IFN-*γ methylation distributions. PM_2.5_ mass concentrations were also negatively related to the mean of the *F3* methylation distribution.

**Table 5:** Associations with mean gene-specific methylation for an interquartile range increase in air pollutant concentration.

	Mean ratio^*a*^ (95% CI)			Mean difference^*b*^ (95% CI)	
	*F3*	*ICAM-1*	*TLR-2*	*IFN*-γ	*IL-6*
Particle number	0.78 (0.72, 0.85)*	0.97 (0.92, 1.03)	1.00 (0.94, 1.08)	–0.77 (–1.43, –0.11)*	0.59 (–0.55, 1.74)
PM_2.5_ black carbon	0.90 (0.85, 0.95)*	0.98 (0.95, 1.02)	1.03 (0.98, 1.08)	–0.41 (–0.87, 0.04)	0.76 (–0.03, 1.54)
PM_2.5_ mass	0.96 (0.93, 1.00)	0.97 (0.94, 1.00)	1.00 (0.97, 1.03)	–0.18 (–0.49, 0.13)	0.33 (–0.19, 0.84)
^***a***^Mean ratio for *F3, ICAM-1*, and *TLR-2* (multiplicative scale): Because the methylation distributions of *F3, ICAM-1*, and *TLR-2* had a point mass at zero and the residuals’ distribution showed important deviation from a Gaussian density, we assumed a Tweedie distribution (with a log-link) for these outcomes and reported associations on the multiplicative scale. ^***b***^Mean difference for *IFN*-γ and *IL-6* (absolute scale): For the other outcome distributions (i.e., *IFN*-γ and *IL-6* methylation), we assumed a Gaussian distribution for the residuals and presented our results on the additive scale. *Significant at *p* = 0.05.					

**Figure 2:**
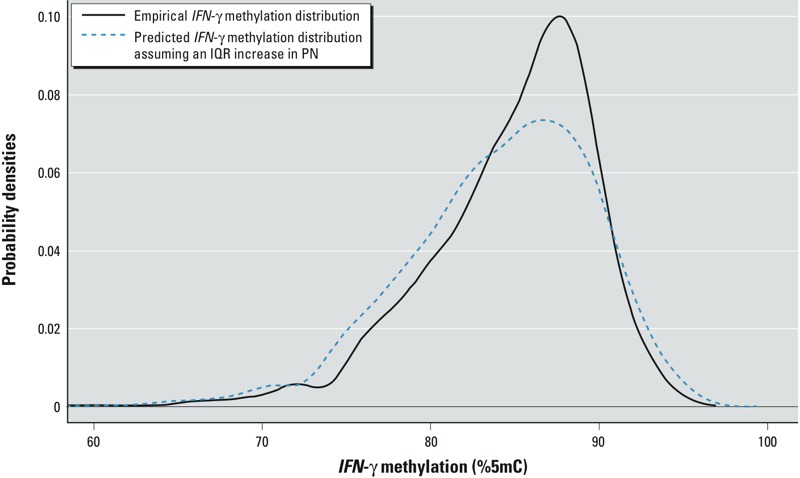
Empirical *IFN-*γ methylation distribution and its associated predicted distribution assuming an IQR increase in particle number concentration. Abbreviations: IQR, interquartile range; PN, particle number.****The results show that instead of air pollution being associated with a shifting of the entire distribution to the left, it is associated with a distortion of its shape, increasing in particular the probabilities of lower methylation levels.

We propose another way of presenting the decile-specific results (i.e., finding reported in Figure 1). We focused on the association between particle number and the *IFN-*γ methylation distribution (presented in third top panel in Figure 1). Particle number was associated with a left shift in the lower tail of the *IFN-*γ methylation distribution (Figure 2).

## Discussion

Our findings suggest a potential impact of air pollution on DNA methylation and heterogeneous associations across quantiles of some gene-specific methylation distributions. In the same cohort, aging has been related to hypomethylation of *TLR-2* and hypermethylation of *F3* and *IFN-*γ (Madrigano et al. 2012b); and compared with never and former smokers, current smokers had higher *IL-6* methylation and lower *TLR-2* and *IFN-*γ methylation levels (Bind et al. 2014). Moreover, when we used mean regression to conduct mediation analyses in the same cohort (with air pollution as exposure, methylation as mediator, and cardiovascular-related blood markers as outcomes), we estimated a positive indirect effect of PM_2.5_ black carbon on fibrinogen through a decrease in *F3* methylation (Bind et al. 2014). Similarly, the positive associations of sulfate and ozone with ICAM-1 seemed to be partly mediated via a decrease in *ICAM-1* methylation. This quantile regression study showed that air pollution may be associated with only one extreme of the methylation distribution—which suggests heterogeneity between study participants with respect to potential epigenetic effects resulting from air pollution exposure.

Our results suggest that exposure to fine and ultrafine particles (size between 0.007 and 3 μm in diameter) is associated with decreased methylation in the upper quantiles of *F3* methylation and the lower quantiles of *IFN-*γ methylation. F3, also known as tissue factor, is a major trigger of the coagulation cascade. F3 expression has been observed in vascular smooth muscle cells, endothelial cells, and fibroblasts (which play a role in wound healing) (Holy and Tanner 2010). High F3 levels found in atherosclerotic plaques have been shown to be critical in the pathogenesis of atherothrombosis (Jude et al. 2005). Individuals with acute coronary syndromes, hypertension, dyslipidemia, diabetes, and cancer also have elevated F3 concentrations (measured, for instance, in endothelial cells, monocytes, macrophages, plasma) compared with individuals free of these diseases (Holy and Tanner 2010; Steffel et al. 2006). Furthermore, F3 induces thrombin formation leading to fibrin generation and activation of platelets (Jude et al. 2005). Platelet activation has, in turn, been observed after exposure to ultrafine particles in 57 men with coronary heart disease (Rückerl et al. 2007). An intermediary mechanism could be through inflammatory cytokines and oxidized lipids which have been shown to up-regulate F3 expression (Holy and Tanner 2010; Jude et al. 2005).

IFN-γ is a cytokine that plays a central role in the generation and release of reactive oxygen species (ROS). The formation of ROS is associated with lack of important antioxidants, which causes oxidative stress (Schroecksnadel et al. 2006). According to the findings of several studies, oxidative stress appears to be an intermediary process between air pollution and cardiovascular disease (Barregard et al. 2008; Li et al. 2009; Mazzoli-Rocha et al. 2010; Schroecksnadel et al. 2006).

In this study, exposures to particle number, PM_2.5_ black carbon, and PM_2.5_ mass were associated with the lowest quantiles of *ICAM-1* methylation. In a previous study, we showed that a decrease in *ICAM-1* methylation was also related to a significant increase in the mean of ICAM-1 protein (Bind et al. 2014). ICAM-1 is a glycoprotein that is expressed on endothelial cells and cells of the immune system. Elevated ICAM-1 concentration increases the risk of myocardial infarction or coronary death. Our results suggest that air pollution exposure may decrease *ICAM-1* methylation, which may result in *ICAM-1* gene de-silencing and ICAM-1 protein overexpression.

Our findings using quantile regression are fairly consistent with mean regression analyses using distributed-lag models (Bind et al. 2014) or moving averages (Table 5) for exposure in the same cohort. Concentrations of particle number and PM_2.5_ black carbon were associated with *F3* hypomethylation in both the mean and quantiles analyses. In the mean regression analysis using moving averages for exposure, concentrations of particle number and PM_2.5_ black carbon were not significantly related to *ICAM-1* methylation. This quantile analysis reveals some association between particle number and the low end of the *ICAM-1* methylation distribution and no change at the high end of the distribution, demonstrating the added value of the quantile regression approach. In addition, for PM_2.5_ black carbon, we observed significant negative associations with the lower percentiles of the *ICAM-1* methylation distribution and a positive association with the 90th percentile, indicating an effect of broadening the distribution at both ends, which resulted in a nonsignificant change on average. Particle number concentration was associated with the lower percentiles and the mean of the *IFN-*γ methylation distribution. However, the magnitude of the mean estimate was smaller compared with the estimates of the lower percentiles. For example, an interquartile range increase in particle number concentration was associated with a 0.8%5mC (95% CI: 0.1, 1.4; Table 5) and a 1.5%5mC (95% CI: 0.6, 2.4; Figure 1) decrease in mean and the 20th percentile of the *IFN-*γ methylation distribution, respectively.

Quantile regression allows us to describe effects that shift the overall shape, as opposed to the location, of the outcome distribution. For instance, although we found some evidence that exposure to fine and ultrafine particles (size between 0.007 and 3 μm in diameter) shifts the low quantiles of the *IFN-*γ methylation distribution toward lower levels, we observed no significant effect on the upper quantiles. Figure 2 shows the distributional change of *IFN-*γ methylation assuming an interquartile range increase in particle number concentration. Our findings suggest that participants with low *IFN-*γ methylation may be more susceptible to fine and ultrafine particles. In our study population of older men, air pollution exposures were associated with a left shift in the lower tail of the *IFN-*γ methylation distribution.

Also, the heterogeneous associations between air pollution and methylation across quantiles of the methylation distribution is seen with mostly particle number for *F3* and *IFN-*γ and is seen with particle number, PM_2.5_ black carbon, and PM_2.5_ mass concentrations for *ICAM-1*. Different types of pollutants and size of particles may therefore have varying effects on gene-specific methylation.

*Method limitations and strengths*. Individuals in the top 50% of the *F3* methylation distribution (i.e., with methylation levels between 2.0 and 4.5%5mC; Table 4) tend to lose about 1%5mC of methylation per IQR increase in particle number (Figure 1), which corresponds to almost double the loss observed in individuals in the bottom 50% (i.e., with methylation levels between 1.0 and 2.0%5mC). Because a given CpG site in a given homozygotic cell is either (fully) methylated or (fully) unmethylated, we acknowledge that the first group of participants has roughly twice as many circulating methylated cells as the second, so the higher impact of exposure in the top 50% group versus the bottom 50% group may not be unexpected. Furthermore, the result is an increase by 1 or 0.5% of the proportion of unmethylated (possibly F3-expressing) cells from a baseline of 95.5–98% and 98–99% (Table 4), respectively, a change whose significance is debatable. On the other hand, for *IFN-*γ methylation the corresponding picture is that, in people with about 75–85%5mC methylation (i.e., only 15–25% of the circulating cells are unmethylated; Table 4), the frequency of unmethylated cells increases by 1% per IQR in PM_2.5_ mass concentration, a sizable and potentially significant increase. However, these arguments could be reversed if what matters physiologically is a large change on the ratio scale compared with the absolute scale. However, data collected in this study population are limited to address this issue.

Quantile regression is a distribution-free method and allows us to obtain estimates on the additive scale (expressed as a change in %5mC). In contrast, the standard approach using mean regression requires assumptions about the distribution of the residuals or the outcome. This approach using quantile regression can be reused in other disciplines to target susceptible population. In the presence of heterogeneity, reporting the exposure–outcome association along the entire outcome distribution could also add some preciseness in estimates used for risk assessment. Taking into account the mean effect on an outcome that is likely to differ according to the quantile of methylation in which participants belong could be misleading. Epidemiological studies reporting associations based on conditional means may miss what is happening in some part of the study population.

## Conclusions

Quantile regression suggested shifts in methylation distributions associated with air pollution exposure that were not captured by corresponding least-square estimates of the difference in (or ratio of) mean methylation associated with exposure. In the case of *ICAM-1* and *IFN-*γ methylation, negative associations between particle number concentration and methylation were concentrated on the lower deciles of the methylation distribution—that is, among individuals who already had low methylation levels, consistent with a shift on the lower quantiles of the methylation distribution to the left. Although the role of methylation in gene expression is complex, including no role, methylation tends to repress expression (Riggs and Xiong 2004). Hence, it is possible that individuals who already had a higher risk of inflammation may be the ones primarily affected by particles. In summary, quantile regression may capture associations that are only in the tails of the distribution and might be otherwise missed. This approach estimating associations along outcome distribution also allows us to describe distributional outcome changes associated with increasing exposure. This makes it a valuable tool for environmental epidemiology, and for providing results that might allow better risk assessment in future studies.

## Supplemental Material

(515 KB) PDF
