# Supplementary material for: Beyond the Mean: Quantile Regression to Explore the Association of Air Pollution with Gene-Specific Methylation in the Normative Aging Study
Source: Environ Health Perspect. 2015 Mar 13;123(8):759–65. doi: 10.1289/ehp.1307824 (PMC4529003; doi:10.1289/ehp.1307824)
Supplement: (515 KB) PDF [file ehp.1307824.s001.acco.pdf]

Environ Health Perspect
doi: 10.1289/ehp.1307824

Note to Readers: ***EHP*** **strives to ensure that all journal content is accessible to all readers. However, some figures and Supplemental Material published in** ***EHP*** **articles may not conform to 508 standards due to the complexity of the information being presented. If you need assistance accessing journal content, please contact ehp508@niehs.nih.gov. Our staff will work with you to assess and meet your accessibility needs within 3 working days.**

Supplemental Material

Beyond the Mean: Quantile Regression to Explore the Association of Air Pollution with Gene-Specific Methylation in the Normative Aging Study

Marie-Abele C. Bind, Brent A. Coull, Annette Peters, Andrea A. Baccarelli, Letizia Tarantini, Laura Cantone, Pantel S. Vokonas, Petros Koutrakis, and Joel D. Schwartz

Table of Contents

Figure S1. Directed Acyclic Graph (DAG) at adjacent visits j=J and j=J+1. This DAG illustrates the relationships we assumed between the variables included in the regression models. A_i^j represents the 4-week moving average of air pollutant concentration before the jth visit of participant i. Y_i^j represents the ith participant gene-specific DNA methylation at visit j. $\psi_p(Y_i^j)$ is the pth quantile of the Y_{ij} distribution. C_{1i}^j and C_{2i}^j correspond to the potential confounding variables and risk factors of DNA methylation for participant i[th] at visit j, respectively. b_{0i} represents the random intercept of participant i.

Figure S2. Sensitivity analyses restricted to never and former smokers: absolute difference in gene-specific methylation (expressed in %5mC with 95%CI) associated with an IQR increase in exposure (IQR=14,599 number per cm^3 for particle number, 0.26 $\mu g/m^3$ for $PM_{2.5}$ black carbon, and 3.4 $\mu g/m^3$ for $PM_{2.5}$ mass), according to deciles of the methylation distribution. In this secondary analysis, we considered only never and former smokers based on the time-varying smoking status variable. The analysis included 755 participants and 1,737 individual observations.

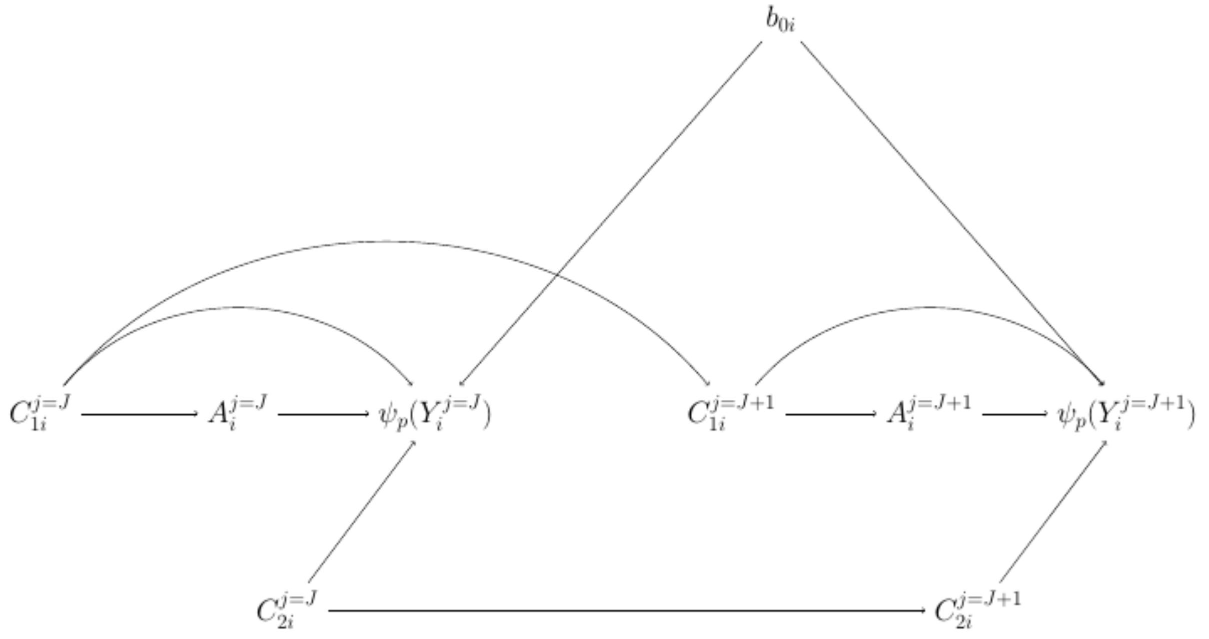

Figure S1. Directed Acyclic Graph (DAG) at adjacent visits j=J and j=J+1. This DAG illustrates the relationships we assumed between the variables included in the regression models. A_i^j represents the 4-week moving average of air pollutant concentration before the jth visit of participant i. Y_i^j represents the ith participant gene-specific DNA methylation at visit j. $\psi_p(Y_i^j)$ is the pth quantile of the Y_{ij} distribution. C_{1i}^j and C_{2i}^j correspond to the potential confounding variables and risk factors of DNA methylation for participant i^{th} at visit j, respectively. b_{0i} represents the random intercept of participant i.

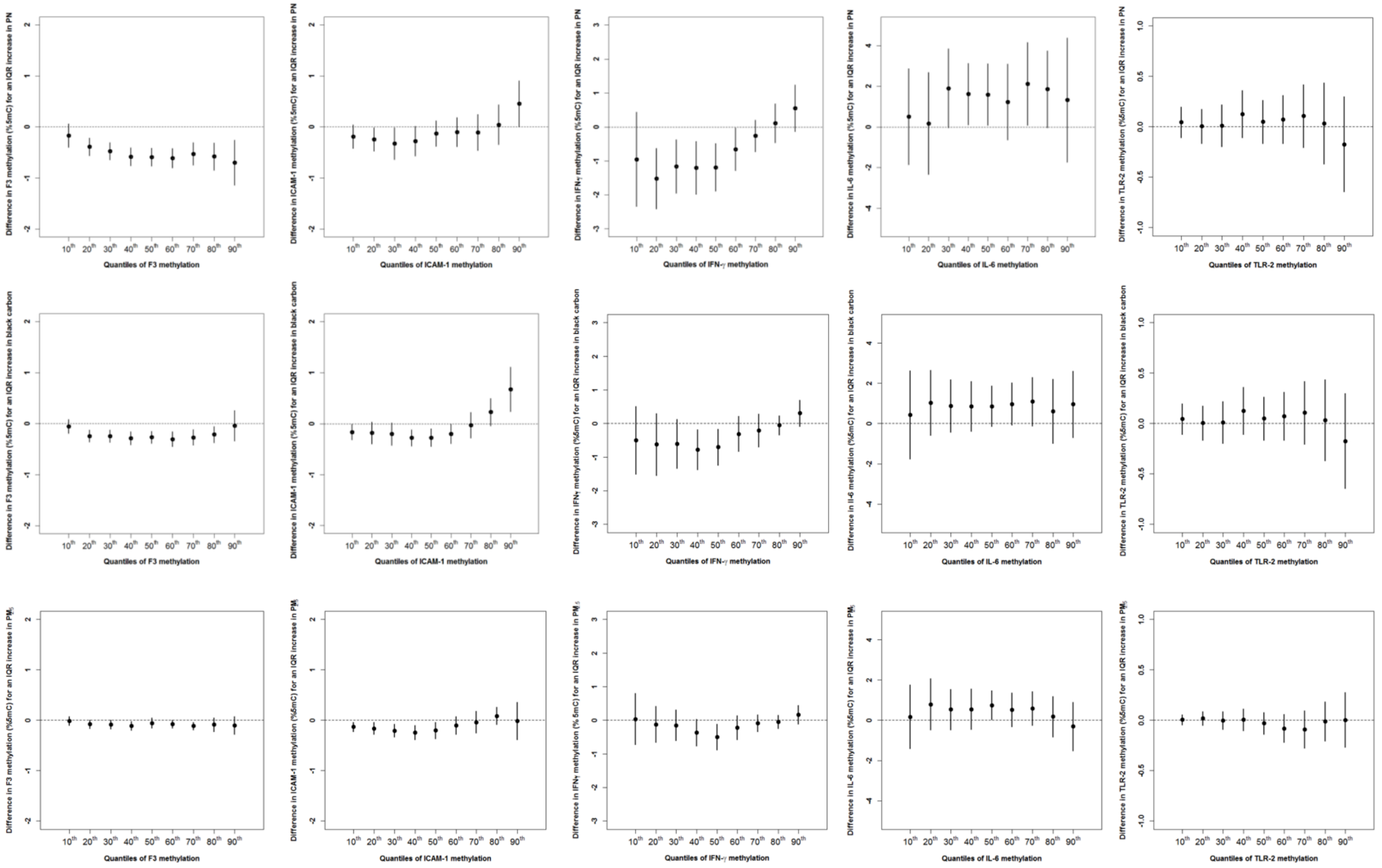

Figure S2. Sensitivity analyses restricted to never and former smokers: absolute difference in gene-specific methylation (expressed in %5mC with 95%CI) associated with an IQR increase in exposure (IQR=14,599 number per cm^3 for particle number, 0.26 μg/m^3 for $PM_{2.5}$ black carbon, and 3.4 μg/m^3 for $PM_{2.5}$ mass), according to deciles of the methylation distribution. In this secondary analysis, we considered only never and former smokers based on the time-varying smoking status variable. The analysis included 755 participants and 1,737 individual observations.
